# Supplementary material for: Climate gradients, and patterns of biodiversity and biotic homogenization in urban residential yards
Source: PLoS One. 2020 Aug 28;15(8):e0234830. doi: 10.1371/journal.pone.0234830 (PMC7454958; doi:10.1371/journal.pone.0234830)
Supplement: S1 Fig — Towns are shown in their relative N-S and E-W orientations. ‘Shells’ included both macro- and micro- snails—and includes some live microsnails, ‘Live snails’ were field-identified snails and slugs that were released on site or reared for identification, ‘‘Soil sample’ included primarily microsnails–mostly shells, but also live snails. (PDF) [file pone.0234830.s001.pdf]

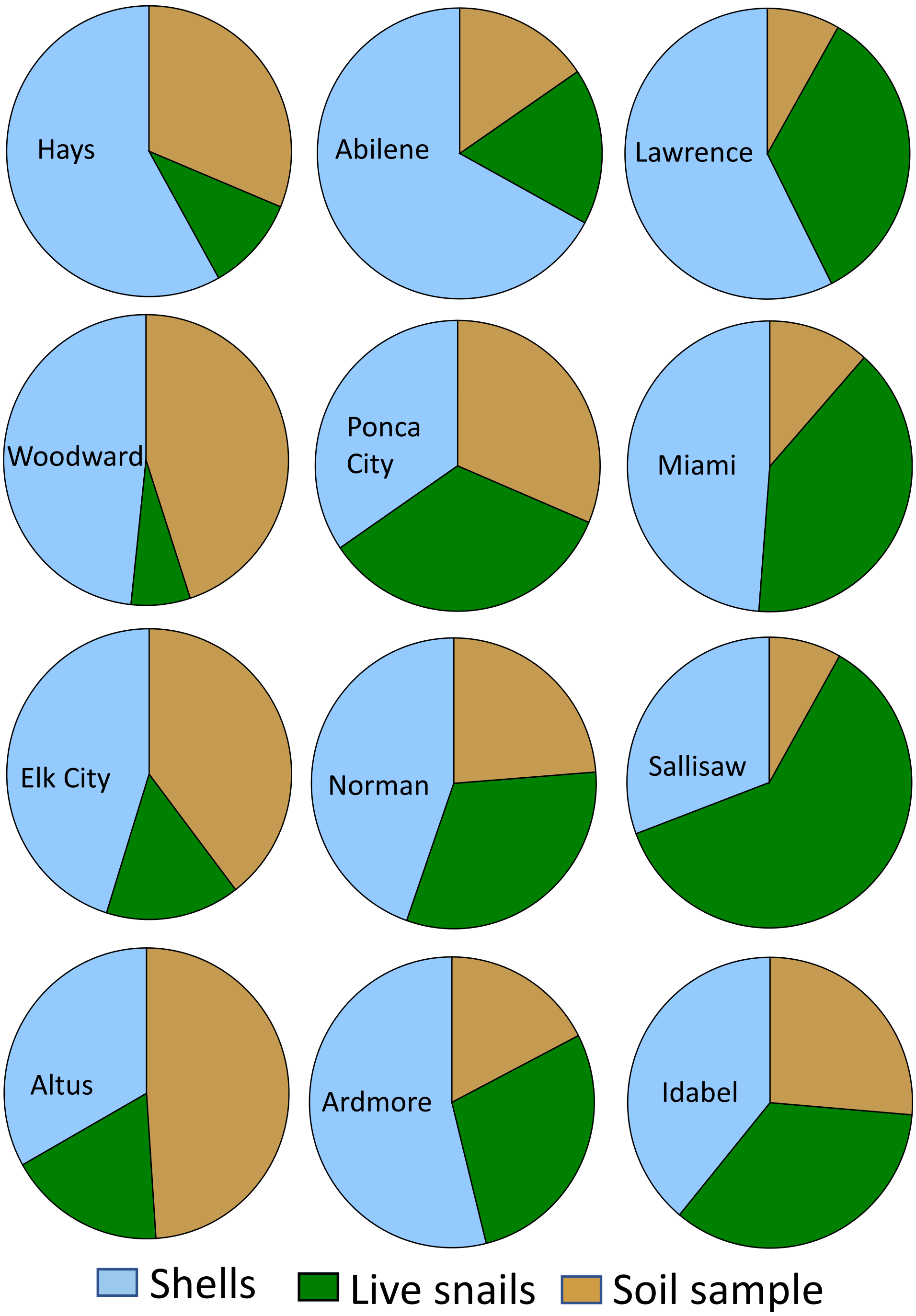

S1 Fig. Percent contribution of each sample type to the total abundance in each of the 12 surveyed towns. Towns are shown in their relative N-S and E-W orientations. 'Shells' included both macro- and micro- snails - and includes some live microsnails, 'Live snails' were field-identified snails and slugs that were released on site or reared for identification, ''Soil sample' included primarily microsnails – mostly shells, but also live snails.
