# Supplementary material for: Climate gradients, and patterns of biodiversity and biotic homogenization in urban residential yards
Source: PLoS One. 2020 Aug 28;15(8):e0234830. doi: 10.1371/journal.pone.0234830 (PMC7454958; doi:10.1371/journal.pone.0234830)
Supplement: S2 Fig — The graph is based on snails counted and released during the visual survey and undercounts live microsnails that were included in the shell or soil samples. (PDF) [file pone.0234830.s002.pdf]

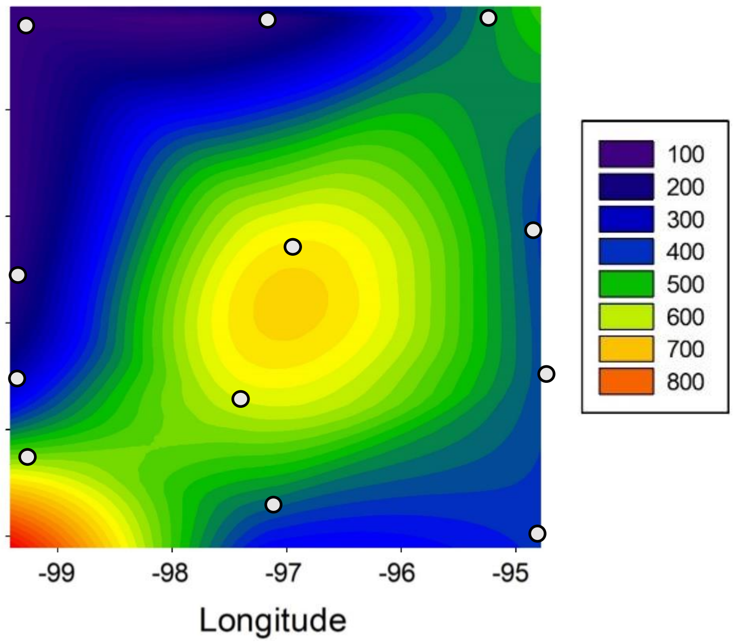

S2 Fig. Spatial pattern of live snail abundance across the 12 surveyed towns. The graph is based on snails counted and released during the visual survey and undercounts live microsnails that were included in the shell or soil samples.
