# Supplementary material for: Climate gradients, and patterns of biodiversity and biotic homogenization in urban residential yards
Source: PLoS One. 2020 Aug 28;15(8):e0234830. doi: 10.1371/journal.pone.0234830 (PMC7454958; doi:10.1371/journal.pone.0234830)
Supplement: S1 Table — Population is based on the US Census Bureau (2018) estimates for 2016. Climatic data are means over a 20-yr period. Latitude and longitude are mean values for the ten yards sampled in each town. (PDF) [file pone.0234830.s003.pdf]

S1 Table. Twelve towns included in the regional survey. Population is based on the US Census Bureau (2018) estimates for 2016. Climatic data are means over a 20-yr period. Latitude and longitude are mean values for the ten yards sampled in each town.

Town	Population	January mean low temp. (°C)	Annual rainfall (cm)	Sampling period	Mean latitude	Mean longitude
Hays, KS	21,027	-8.4	61.3	12-14 Jun 2018	38.88306	-99.3127
Abilene, KS	6,469	-5.6	81.7	30-31 May 2018	38.92687	-97.2156
Lawrence, KS	95,358	-7.8	85.0	28-29 May 2018	38.95647	-95.2614
Woodward, OK	12,543	-3.7	64.1	14-16 Jun 2017	36.42862	-99.4078
Ponca City, OK	24,527	-4.6	78.4	28 Apr-7 May 2017	36.71878	-97.0618
Miami, OK	13,484	-3.6	108.1	22-24 May 2018	36.88507	-94.8689
Elk City, OK	11,997	-4.2	75.0	14-15 May 2018	35.41175	-99.4133
Norman, OK	122,180	-2.5	93.2	1-23 Jun 2017	35.21593	-97.4494
Sallisaw, OK	8,602	-2.0	111.2	21-22 May 2018	35.46195	-94.783
Altus, OK	19,163	-2.5	67.4	5-7 Jun 2017	34.66462	-99.3295
Ardmore, OK	25,107	-0.6	96.9	7-9 May 2018	34.17906	-97.1427
Idabel, OK	6,950	0.1	113.8	27-29 Jun 2017	33.88793	-94.8109
