# Supplementary material for: Climate gradients, and patterns of biodiversity and biotic homogenization in urban residential yards
Source: PLoS One. 2020 Aug 28;15(8):e0234830. doi: 10.1371/journal.pone.0234830 (PMC7454958; doi:10.1371/journal.pone.0234830)
Supplement: S2 Table — (PDF) [file pone.0234830.s004.pdf]

S2 Table. Taxa list, native/non-native designation relative to Oklahoma and Kansas, and total count of each taxon for the 10 yards in the 12 towns in the survey.

Family - taxon	Nativity	Lawrence	Abilene	Hays	Woodward	Ponca City	Miami
Agriolimacidae							
Deroceras laeve	native	8	10	4	5	19	218
Deroceras reticulatum	alien	0	0	0	0	1	0
Agriolimacidae							
unidentified slug	?	15	0	1	0	0	0
Arionidae							
Arion sp.	alien	0	0	0	0	0	0
Bradybaenidae							
Bradybaena similaris	alien	0	0	0	111	0	0
Cionellidae							
Cochlicopa lubricella	native	1	0	0	2	0	0
Discidae							
Anguispira alternata	native	18	0	0	0	226	0
Helicarionidae							
Euconulus trochulus	native	0	0	0	0	0	1
Helicidae							
Cornu aspersum	alien	0	0	0	0	0	0
Helicinidae							
Oligyra orbiculata	native	0	0	0	0	0	0
Helicodiscidae							
Helicodiscus parallelus	native	1	3	1	0	6	2
Limacidae							
Ambigolimax valentianus	alien	0	1	0	0	52	26
Limax flavus	alien	0	0	0	0	0	0
Limax maximus	alien	0	1	0	0	0	7
Milacidae							
Milax gagates	alien	0	0	0	0	19	0

Polygyridae							
Daedalochila leporina	native	0	0	0	0	0	0
Euchemotrema leai alicae	native	0	5	0	0	0	0
Mesodon thyroides	native	322	63	0	28	19	7
Patera appressa	extralimital	0	0	0	0	20	0
Polygyra cereolus	extralimital	0	0	0	21	0	1
Linisia texasiana	native	0	0	0	30	72	5
Triodopsis hopetonensis	extralimital	60	16	0	123	251	183
Xylotrema fosteri	extralimital	149	27	0	0	2	0
Vertiginidae							
Gastrocopta armifera	native	17	13	34	81	0	11
Gastrocopta contracta	native	19	24	12	52	174	13
Gastrocopta cristata	native	1	5	72	237	0	0
Gastrocopta pellucida	native	0	0	0	55	4	0
Gastrocopta pentodon	native	0	1	0	1	0	23
Gastrocopta procera	native	4	7	67	4	35	0
Gastrocopta sterkiana	native	3	0	0	19	1	38
Gastrocopta tappaniana	native	0	0	0	2	2	6
Vertigo gouldii	extralimital	2	0	0	0	0	0
Vertigo milium	native	0	0	0	0	1	1
Vertigo rugosula	native	0	0	0	0	1	2
Vertigo teskeyae	extralimital	0	0	0	0	0	0
Pupillidae							
Pupoides albilabris	native	7	12	65	138	7	10
Vertiginidae							
Columella simplex	native	0	0	0	0	0	0
Stropilopsidae							
Strobilops sp.	native	26	1	0	2	2	0
Subulinidae							
Allopeas clavulinum	alien	0	0	0	0	0	0
Opeas pyrgula	alien	133	0	0	33	35	5
Rumina decollata	alien	0	0	0	0	0	0

Succineidae							
unidentified	?	0	6	3	189	5	50
Vallonidae							
Vallonia pulchella	extralimital	109	117	558	49	0	5
Vallonia parvula	extralimital	6	1	31	74	0	2
Zonitidae							
Glyphyalinia indentata	native	57	14	0	0	127	19
Hawaiia minuscula	native	53	104	64	352	268	138
Nesovitrea electrina	native	1	0	0	0	0	0
Ventridens demissus	extralimital	118	0	0	0	307	51
Zonitoides arboreus	native	129	118	60	361	179	194
Unidentified							
(immatures)	native	50	19	17	84	68	13

S2 Table (continued).

**Family - taxon**	**Nativity**	**Elk City**	**Norman**	**Sallisaw**	**Altus**	**Ardmore**	**Idabel**
Agriolimacidae							
Deroceras laeve	native	4	3	69	21	13	2
Deroceras reticulatum	alien	0	0	0	0	0	0
Agriolimacidae							
unidentified slug	?	0	0	0	0	0	0
Arionidae							
Arion sp.	alien	0	0	1	0	0	0
Bradybaenidae							
Bradybaena similaris	alien	0	0	0	0	47	0
Cionellidae							
Cochlicopa lubricella	native	0	0	0	0	0	0
Discidae							
Anguispira alternata	native	0	122	0	0	0	0

Helicarionidae							
Euconulus trochulus	native	0	0	0	0	0	0
Helicidae							
Cornu aspersum	alien	0	0	0	9	0	0
Helicinidae							
Oligyra orbiculata	native	0	0	0	0	0	36
Helicodiscidae							
Helicodiscus parallelus	native	0	0	3	0	16	0
Limacidae							
Ambigolimax valentianus	alien	1	3	12	22	2	7
Limax flavus	alien	0	0	5	4	2	0
Limax maximus	alien	0	0	0	0	0	0
Milacidae							
Milax gagates	alien	0	0	0	0	0	0
Polygyridae							
Daedalochila leporina	native	0	0	0	0	0	5
Euchemotrema leai alicae	native	0	0	0	0	0	0
Mesodon thyroides	native	0	9	0	1	8	0
Patera appressa	extralimital	0	0	0	0	0	0
Polygyra cereolus	extralimital	30	0	0	592	52	0
Linisia texasiana	native	149	52	20	117	24	32
Triodopsis hopetonensis	extralimital	0	128	168	98	208	133
Xylotrema fosteri	extralimital	0	3	0	0	0	0
Vertiginidae							
Gastrocopta armifera	native	41	9	0	0	0	2
Gastrocopta contracta	native	59	85	0	83	61	22
Gastrocopta cristata	native	252	9	0	201	21	0
Gastrocopta pellucida	native	23	22	0	99	19	3
Gastrocopta pentodon	native	0	3	0	102	2	25
Gastrocopta procera	native	6	2	0	2	0	0
Gastrocopta sterkiana	native	0	12	10	78	2	2
Gastrocopta tappaniana	native	15	0	2	5	1	0

Vertigo gouldii	extralimital	0	0	0	0	0	0
Vertigo milium	native	0	0	0	0	0	0
Vertigo rugosula	native	0	7	6	0	32	11
Vertigo teskeyae	extralimital	0	0	2	0	0	0
Pupillidae							
Pupoides albilabris	native	144	52	12	72	34	3
Vertiginidae							
Columella simplex	native	3	0	0	0	0	0
Stropilopsidae							
Strobilops sp.	native	0	16	0	37	58	38
Subulinidae							
Allopeas clavulinum	alien	0	0	0	0	2	0
Opeas pyrgula	alien	0	27	55	51	74	60
Rumina decollata	alien	0	0	0	100	19	0
Succineidae							
unidentified	?	277	0	6	53	18	5
Vallonidae							
Vallonia pulchella	extralimital	106	0	0	3	0	0
Vallonia parvula	extralimital	0	0	0	0	0	0
Zonitidae							
Glyphyalinia indentata	native	4	47	3	1	90	10
Hawaiia minuscula	native	291	347	50	977	152	241
Nesovitrea electrina	native	0	0	0	0	0	0
Ventridens demissus	extralimital	0	761	188	14	204	329
Zonitoides arboreus	native	415	65	44	282	169	100
Unidentified							
(immatures)	native	86	84	14	266	71	43
