# Supplementary material for: Climate gradients, and patterns of biodiversity and biotic homogenization in urban residential yards
Source: PLoS One. 2020 Aug 28;15(8):e0234830. doi: 10.1371/journal.pone.0234830 (PMC7454958; doi:10.1371/journal.pone.0234830)
Supplement: S3 Table — (PDF) [file pone.0234830.s005.pdf]

S3 Table. Pairwise comparison results following PERMANOVA test of the Bray Curtis similarity of the yard-dwelling land snail populations in 12 towns, which produced 66 pair-wise comparisons.

Town pairs	t	P	Unique permutations
Ponca, Norman	1.2538	0.102	993
Ponca, Altus	2.2403	0.001	995
Ponca, Woodward	1.952	0.001	989
Ponca, Idabel	1.2795	0.086	993
Ponca, Ardmore	1.083	0.266	993
Ponca, Elk City	2.0734	0.001	989
Ponca, Sallisaw	1.6629	0.006	990
Ponca, Miami	1.8246	0.003	988
Ponca, Lawrence	2.3597	0.001	995
Ponca, Abilene	2.0552	0.001	994
Ponca, Hays	2.5379	0.001	988
Norman, Altus	2.2278	0.003	988
Norman, Woodward	2.0875	0.001	997
Norman, Idabel	1.4234	0.074	997
Norman, Ardmore	1.3659	0.07	994
Norman, Elk City	2.1549	0.001	989
Norman, Sallisaw	1.8437	0.003	992
Norman, Miami	2.359	0.001	994
Norman, Lawrence	2.5852	0.001	994
Norman, Abilene	2.3025	0.001	990
Norman, Hays	2.6017	0.001	995
Altus, Woodward	1.8385	0.001	994
Altus, Idabel	2.7816	0.001	995
Altus, Ardmore	2.2843	0.001	996
Altus, Elk City	1.8911	0.001	990
Altus, Sallisaw	2.9681	0.001	994

Altus, Miami	2.8421	0.001	991
Altus, Lawrence	3.4052	0.001	992
Altus, Abilene	2.8323	0.001	988
Altus, Hays	3.0051	0.001	994
Woodward, Idabel	2.5138	0.001	994
Woodward, Ardmore	1.7096	0.002	992
Woodward, Elk City	0.86734	0.65	993
Woodward, Sallisaw	2.5484	0.001	994
Woodward, Miami	2.1806	0.001	995
Woodward, Lawrence	2.7627	0.001	994
Woodward, Abilene	2.1937	0.001	989
Woodward, Hays	2.1432	0.001	989
Idabel, Ardmore	1.2981	0.079	995
Idabel, Elk City	2.605	0.001	993
Idabel, Sallisaw	1.45	0.023	992
Idabel, Miami	2.319	0.001	991
Idabel, Lawrence	2.9776	0.001	987
Idabel, Abilene	2.4837	0.001	996
Idabel, Hays	3.0236	0.001	987
Ardmore, Elk City	1.9807	0.001	993
Ardmore, Sallisaw	1.6346	0.007	991
Ardmore, Miami	1.726	0.005	991
Ardmore, Lawrence	2.3439	0.001	998
Ardmore, Abilene	2.0199	0.001	991
Ardmore, Hays	2.5602	0.001	991
Elk City, Sallisaw	2.6767	0.001	995
Elk City, Miami	2.3286	0.001	995
Elk City, Lawrence	2.8147	0.001	997
Elk City, Abilene	1.9513	0.001	997
Elk City, Hays	1.905	0.001	996
Sallisaw, Miami	1.8725	0.002	995

Sallisaw, Lawrence	2.7897	0.001	990
Sallisaw, Abilene	2.2449	0.001	993
Sallisaw, Hays	2.6746	0.001	989
Miami, Lawrence	2.8595	0.001	992
Miami, Abilene	2.1688	0.001	994
Miami, Hays	2.8277	0.001	994
Lawrence, Abilene	2.1209	0.001	996
Lawrence, Hays	2.7218	0.001	993
Abilene, Hays	1.5918	0.015	994

(PERMANOVA: Pseudo-$F_{11,108}$ = 5.0955 P = 0.001). Pair-wise testing used 999 permutations.
